# Usability and value of a digital learning resource in nursing education across European countries: a cross-sectional exploration

**DOI:** 10.1186/s12912-021-00681-5

**Published:** 2021-09-06

**Authors:** Kristin Hjorthaug Urstad, Esther Navarro-Illana, Bjørg Oftedal, Katharine Whittingham, Santiago Alamar, Richard Windle, Atle Løkken, Michael Taylor, Marie Hamilton Larsen, Melanie Narayasanamy, Javier Sancho-Pelluz, Pedro Navarro-Illana, Heather Wharrad

**Affiliations:** 1 Faculty of Health Sciences, University of Stavanger, 4036 Stavanger, Norway; 2 Faculty of Health Studies, VID Specialized University, Mailbox 184 Vinderen, NO-0319 Oslo, Norway; 3 The Faculty of Nursing, Valencia Catholic University, Catholic Carrer de Quevedo 2, Valencian Community, 46001 Valencia, Spain; 4 School of Health Sciences, University of Nottingham, Queen’s Medical Centre, NG7 2HA Nottingham, UK; 5 Lovisenberg Diaconal University College, Lovisenberggata 15B, 0456 Oslo, Norway; 6 Institute of Basic Medical Sciences, University of Oslo, P.O box 1130, Blindern, 0318 Oslo, Norway

**Keywords:** E-learning, Health education, Internationalization, Descriptive cross-sectional research design, Undergraduate nursing students

## Abstract

**Background:**

Higher education is responsible for providing education that meets international benchmarks relevant to the needs of the international community. Due to the increase of digital tools in higher education, the possibility of sharing learning resources across nations has expanded. In the current project, a Norwegian university invited universities in Spain and the United Kingdom to adapt and translate e-learning resources originally developed for Norwegian nursing students for use within their respective Bachelor in Nursing programmes.

**Aim:**

The aim of the current study was to gain insights into the usability and value for learning of e-compendiums shared and implemented across three European universities.

**Methods:**

The study adopted a descriptive cross-sectional design and included nursing students from the University of Nottingham, Valencia Catholic University, and the University of Stavanger. Data were collected in Autumn 2017 through a questionnaire adapted from the validated “Centre for Excellence in Teaching and Learning Reusable Learning Object evaluation questionnaire” The questionnaire consisted of 19 items that included two aspects: e-compendiums’ value for learning and e-compendiums’ usability. The different study sites were compared using a binary logistic regression analysis. Subgroups of students were compared based on their gender and age.

**Results:**

A total of 480 nursing students participated in the study. The e -compendiums were overall positively rated, especially for reinforcing and retaining knowledge. Compared to the students from the University of Stavanger, students from Valencia Catholic University rated the e-compendiums more positively in most aspects of learning. Students from University of Nottingham found the e-compendiums to be more important for learning engagement compared to students at the Norwegian study site, and no differences were found in any other aspects of learning. Younger students rated the interactivity and visual components as more important compared to older students.

**Conclusions:**

Students from the University of Nottingham and Valencia Catholic University seem to accept the e-compendiums despite the fact that they were originally developed for use in another country. We argue that, when sharing e-learning resources across countries, an adaptation and translation process that includes a multicultural and multidisciplinary perspective should be carried out.

**Supplementary Information:**

The online version contains supplementary material available at 10.1186/s12912-021-00681-5.

## Background

The renewed European Commission’s agenda for higher education includes incorporating e-learning resources in teaching and in recent years, the global use of digital learning tools has expanded [1, 2]. In the context of health education, recent systematic reviews regarding e-learning reports evidence for the importance of digital pedagogical approaches for achieving desired learners’ outcomes in practice [3, 4]. For nurse education specifically, methods such as blended learning and mobile technology have increasingly been implemented and valued as a positive contribution to learning [4–8]. and a recent published study of nursing student’s experiences during the Covid-19 pandemic showed that except for the negative factor of desocialisation, students appreciated the shift to a more digitalized learning approach [9]. Nursing students report to appreciate the flexibility provided by e-learning tools as they make them able to access information immediately without being restricted to a particular time or location, especially in clinical settings. Further they express that e-learning impacts on their empowerment and feelings of being in control of their learning situation [5, 10].

In line with the sustainable development goals for higher education, internationalization is at the core of universities’ efforts to provide quality in teaching [11]. Higher education has a responsibility with regard to broadening the understanding of societies and cultures in other regions as well as providing an education that meets international benchmarks and standards and is relevant to the needs of the international community. One goal of the Bologna Declaration is the cohesion of European nursing education [12]. Increasing transparency and mutual recognition of competence is important in health education settings [12].

Due to the increase of digital tools in higher education, possibilities of internationalization have expanded and haring learning resources across nations is more convenient than before. The current study is based on an Erasmus+-funded international partnership project, where the overall goal was to contribute to changing European pedagogy towards a modernized, digital, interactive education program as well as contribute to transparency and cohesion of nursing education. Specifically, the University of Stavanger (UoS) in Norway invited the University of Nottingham (UON) in the United Kingdom (UK) and Valencia Catholic University (VCU) in Spain to adapt and translate e-learning resources originally developed in Norway for use in the Bachelor of Nursing programmes within their respective countries. By exploiting the best of consumer technology, developing, and implementing the educational resources for nursing students in a transnational context, the project aimed to increase the quality of both the content of the educational resources as well as students’ learning and flexibility in their mode of study.

However, internationalisation in higher education entails a number of cross-cultural challenges. Traditions of health care differ across cultures [13–15], and courses and subjects must be relevant and sensitive to the educational and legal contexts of the countries in which they are delivered. Therefore, it is important to gain insight into whether digital pedagogical tools are appropriate when being shared. Hence, the current study aimed to explore whether European nursing students in other countries accepted e-compendiums developed in the context of a nursing course in Norway by investigating value for learning and usability of the e-compendiums in students across the three different institutions.

## Methods

### Study context

The study is based on an Erasmus + project in collaboration with the UoS in Norway, the UoN in the UK and the VCU in Spain (Erasmus + Strategic Partnership 2014 – Project no. 2014-1-N001-KA203-000432). All three universities are established educational institutions offering bachelor programmes in nursing. Nursing education at UoS is organized under the Faculty of Health Sciences and enrols approximately 900 nursing students. UoN’s nursing education is organized under the School of Health Sciences (SoHS) and has an average of 250 undergraduate nursing students in each year of the programme. The Faculty of Nursing at VCU is the largest in Spain, with approximately 1,600 nursing students.

### Adaptation process and e-compendiums

A cross-cultural, interdisciplinary project group consisting of nurse educators, learning technologists, researchers, and nursing students collaborated on the translation and adaptation of the e-compendiums. The group organized the activities through three main phases described as iterative ‘flexible loops’ interacting with each other: learning material adaptation, technical development and user feedback [16].

The working methods included face-to-face meetings at the three study sites, workshops, virtual meetings, and a shared working platform (Google Docs), where documents were visible for all group members. As a first step in the process, workshops were held to determine which subjects should be included in the e-compendiums based on what was regarded as relevant and useful in all three education programs. Consensus was through discussion finally reached on the inclusion of eight subjects: four e-compendiums had a specific nurse care focus and four had a biology focus.

Existing Norwegian learning materials were translated into English and Spanish. During workshops and discussions, content was adapted and adjusted to make it relevant and sensitive to the curricula of nurse education in the partner universities. Differences between the partner programs and cultural traditions of nursing care were identified and content quality sought according to each country’s guidelines [17].

The technology platform was changed from PDF and Flash player, and the e-compendiums were redesigned to platform-independent interactive applications based on HTML5 technology and the WordPress editor, which is usable on personal computers as well as smartphones, both online and offline. The interactive materials included simulation games, images, exercises, and self-testing elements. An audio version of the text was recorded and made available both in the app and uploaded separately as podcasts through iTunesU and other channels [18]. (see illustrations found in supplementary files).

Student nurse representatives were included in all development phases of the project to ensure inclusion of the users’ perspective. A beta version of the preliminary e-compendiums was presented to a small group of nursing students from all three countries (*n* = 15). Feedback from the students provided through focus group interviews and by their presence in project meetings guided further development of the product.

**Fig. 1:**
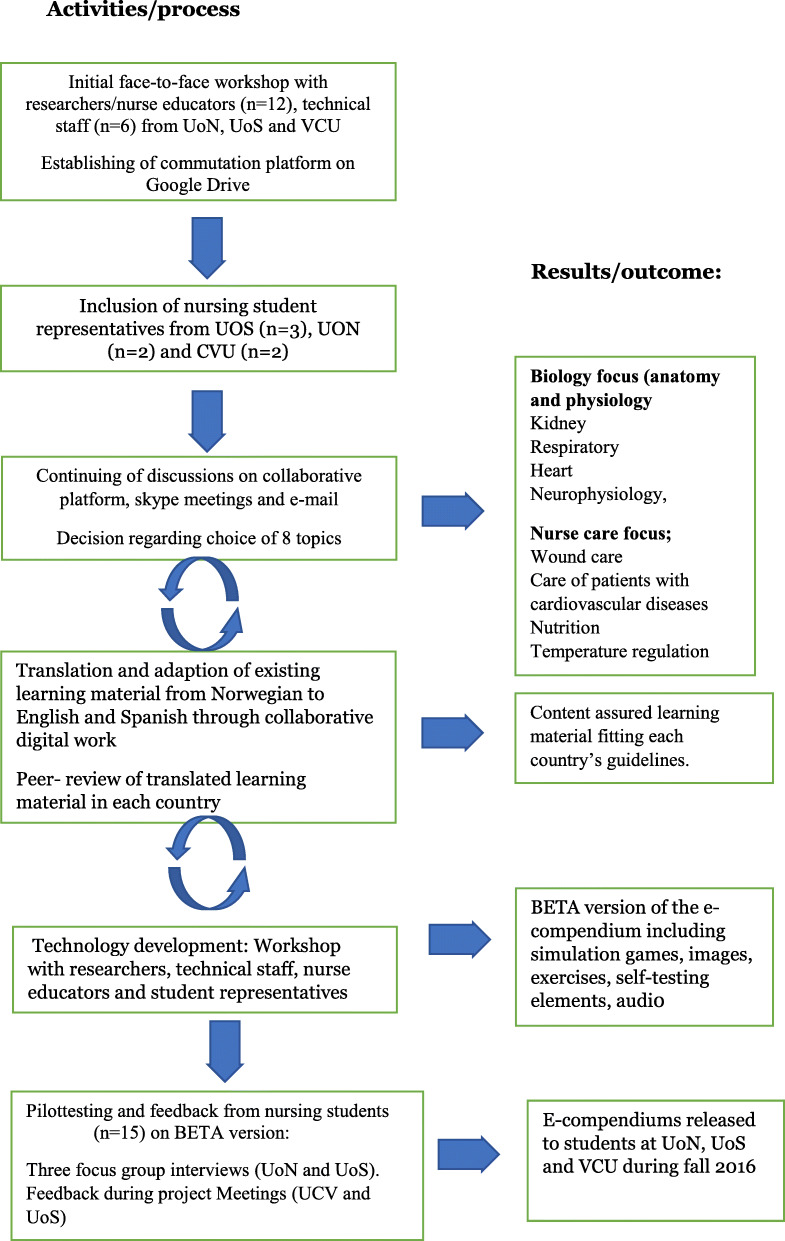
Flow chart outlining key steps in the developing and adaption of the e-compendiums

The e-compendiums were launched to the students in relevant courses at all three study sites during autumn 2016 and spring 2017. The students were informed that the e-compendiums could be used according to individual needs, whether before, during, or after the course. A flow chart outlining key steps in the developing and adaption of the e-compendiums is found in Fig. 1.

### Questionnaire

The questionnaire used for the current study was an adapted version of the “Centre for Excellence in Teaching and Learning Reusable Learning Object evaluation questionnaire” (RLO-CETL) which has previously been utilised to evaluate re-usable learning objects (RLOs) covering a range of different topics [19, 20]. The questionnaire was considered feasible as the e-compendiums had many attributes similar to those of RLOs. However, to ensure more sensitivity and relevance to the current learning resource, the questionnaire was adapted based on in-depth discussions with the transnational project group, which included researchers, lecturers, and technical e-learning experts.

The adapted questionnaire contained 19 items focused on two aspects: values for learning (10 items), rated on a four-point Likert scale anchored on the left with “strongly agree” and on the right with “strongly disagree”, and usability (9 items), rated on a four-point Likert scale anchored on the left with “very useful” and on the right with “not at all useful”. The questionnaire was translated into Norwegian and Spanish by a bilingual speaker. The meaning of every item was critically reviewed by the translation group, and back-translation was carried out. Cronbach’s alphas for the 10 value for learning and 9 usability items were 0.88 and 0.75 respectively.

To detect baseline differences, the questionnaire included an item regarding the usefulness of e-learning as a pedagogical approach in general, rated on a five-point Likert scale anchored on the left with “very useful” and on the right with “not useful at all”. In addition, the students were asked about their confidence in utilizing various e-learning devices for learning (laptop, smartphone, or iPad), which was rated on a five-point Likert scale anchored on the left with “very confident” and on the right with “not confident at all”.

Relevant background characteristics were also collected, such as gender, age, study semester, and full-time or distance students.

### Recruitment

The study utilized a purposive sample technique. The invitation to participate in the study was provided to the students in the semesters in which the 8 e-compendium topics were regarded most relevant. At all three universities, students were informed about the study and asked to participate in relation to a scheduled lecture. At UoS, 152 out of 197 s-semester students attending the lecture agreed to participate (response rate 77 %). At UoN, 49 of 60 third semester students attending the lecture agreed to participate (response rate 82 %). At VCU, first- and second-year students enrolled in a second-semester courses related to the topics included in the e-compendiums were invited to participate during class sessions; of the 320 invited, 279 agreed to participate (response rate 87 %).

### Ethics

All participants were informed about anonymity, confidentiality, publication, their right to withdraw from the study at any time without any consequences and that participating in the study had no impact on their current training.

The study was assessed and approved according to national guidelines in each country. In Norway, approval was given by the Norwegian Centre of Research Data (project number 51,037.). In Spain, the study was approved by the Research and Ethics Committee of VCU (reference 301,117). In the UK, it was approved by the Faculty of Medicine and Health Sciences Ethics Committee on 30 May 2017 (Ethics Reference Number H14112016).

### Analysis

The questionnaire data from the three study sites were exported into the Statistical Package for Social Sciences (SPSS) version 25 (IBM SPSS, Inc., Chicago, IL) using standardized entry codes. For all tests, statistical significance was set at *p* < 05. Descriptive statistics (i.e., gender, age, and school affiliation) were used to present characteristics of the study population. Subgroups of students were compared based on these background characteristics. Student’s *t* test and the chi-squared statistics were used to test for statistical significance.

The different study sites were compared using a binary logistic regression analysis adjusting for gender and age. Variables were recoded from a four-point scale to dichotomous variables. Reference study site was UoS as the aim was to explore how the e-compendiums were accepted by nursing students from UoN and VCU compared to UoS where the original e-compendiums originated.

## Results

### Sample

Of the total sample of 480, the majority were between 18 and 21 years old (63 %) and female (82 %). Participants’ sex and age specified by the three institutions are shown in Table 1.

**Table 1:** Overview of participants from the three educational institutions

	University of Stavanger (*n* = 152)	University of Nottingham (*n* = 49)	Valencia Catholic University (*n* = 279)	Total (*n* = 480)
Sex female	131 (87)	41 (84)	227 (81)	399 (83)
Age:	Valid 151	Valid 47	Valid 279	Valid 477
18–21	80 (52)	19 (40)	202 (72)	301 (63)
22–25	33 (22)	7 (14)	52 (19)	92 (19)
26–35	30 (19)	11 (23)	21 (8)	62 (13)
36–50	8 (5)	10 (21)	4 (1)	22 (5)

Results are given as n (%)

Participants were asked to rate their level of confidence from 1 to 5 (very confident, confident, fairly confident, slightly confident, not confident at all) when utilising different devices for learning. In total, 92 % of students rated themselves as confident or very confident when utilising a laptop for learning. Furthermore, 83 % rated themselves as confident or very confident when using smartphones while 77 % reported being confident or very confident when utilising an iPad for learning. There were no significant differences among the three institutions in terms of confidence when using different devices for learning or in relation to gender or age.

### Value for learning and usability for the total group

The ability to reinforce and retain knowledge was regarded as the most valuable outcome of using the e-compendiums in relation to learning. In total, 95 and 94 % of the total sample agreed or strongly agreed, respectively, that this was the case. Lowest score was found for the value of applying new knowledge; 69 % of all participants agreed or strongly agreed with this statement. Regarding affective statements, 91 % of the students agreed or strongly agreed that the e-compendiums affected their confidence whereas 76 % agreed or strongly agreed that using the e-compendiums made them enjoy learning.

Concerning usability, statements regarding flexibility received highest scores, as 97–98 % of the students reported that being able to reuse the e-compendiums, access them anywhere and anytime, work independently and at own speed, were important or very important. Visual elements were rated as important or very important by 98 % of the students whereas 88 % stated this to be the case for interactive elements in the e-compendiums. The ability to download the material was rated as the least important, as 36 % rated this to be not very important or not at all important. An overview of students’ scores of value in learning and usability is shown in Table 2.

**Table 2:** Participants’ ratings of e-compendiums for learning and usability

	UoS(%)	UoSTotal n	UoNn (%)	UoNTotal n	VCUn (%)	VCUTotal n	All studentsn (%)	All studentsTotal n
**Agree or strongly agree that the e-compendiums were useful for**:								
Being introduced to the topic	137 (91)	145	43 (87)	48	169 (49)	276	349 (74)	470
Reinforcing my knowledge	133 (91)	145	45 (93)	48	268 (97)	277	449 (95)	472
Retaining my knowledge	131 (83)	143	43 (89)	48	268 (96)	278	442 (94)	469
Focusing on essential parts	110 (77)	142	39 (78)	49	242 (88)	276	391 (84)	467
Meeting the requirement of the course	115 (78)	144	39 (78)	49	247 (88)	279	401 (85)	472
Self-assessment	106 (85)	125	35 (71)	49	251 (89)	279	392 (87)	453
Applying new knowledge	125 (85)	146	40 (83)	48	159 (57)	277	324 (69)	471
Increasing my confidence	129 (83)	144	42 (86)	48	259 (93)	278	430 (91)	470
Increasing my motivation	104 (72)	144	37 (77)	48	240 (87)	277	381 (81)	469
Enjoyment of learning	77 (54)	143	31 (67)	46	248 (89)	279	356 (76)	468
**Important or very important for the usability of the e-compendiums**:								
Being able to access learning material anytime	117 (95)	126	41 (95)	43	271 (99)	273	429 (97)	442
Being able to access learning material anywhere	119 (96)	125	41(98)	42	270 (99)	272	430 (97)	439
Being able to work on my own	115 (94)	123	41(95)	43	266 (98)	272	422 (97)	437
Being able to reuse the material	118 (96)	124	42 (95)	44	272 (99)	273	432 (98)	441
Being able to work at my own speed	121 (96)	126	43 (98)	43	268 (98)	272	432 (98)	441
Visual elements	120 (96)	126	40 (93)	43	273 (99)	275	433 (98)	444
Interactive elements	82 (72)	116	41(93)	44	259 (94)	274	382 (88)	435
Download possibility	111 (93)	119	32(76)	42	134 (49)	272	277 (64)	434
Audio elements	76 (61)	126	33(80)	41	199 (72)	274	307 (70)	441

Abbreviations: *UoN* University of Stavanger, *UoN* University of Nottingham, *VUC* Valencia Catholic University

In the total group, younger students (under 26 years old) scored significantly higher than older students for the importance of visual and interactive elements (*p* < 0.001). Furthermore, older students (above 26 years old) rated the ability to download content as more important than younger students did (*p* < 0.001). Female students scored higher on the importance of visual, audio, and downloading possibilities as well as being able to work independently and at own speed compared to male students. Table 3 provides an overview of significant differences between genders in relation to the usability of the e-compendiums.

**Table 3:** Comparison of male and female participants’ ratings for e-compendiums’ different usability elements

	Male	Female	χ^2^ (*p*-value)
Visual elements	70 (87)	363 (95)	7.29 (*p* = 0.007)
Audio elements	39 (48)	269 (72)	17.29 (*p* = 0.001)
Download possibilities	33 (52)	244(68)	6.31 (*p* = 0.015)
Working on my own	64 (91)	358 (98)	6.61 (*p* = 0.012)
Work at my own speed	70 (94)	362 (99)	5.59 (*p* = 0.018)

Results are given as n (%) rating the e-compendium elements as important/very important for usability

### Differences across institutions

Compared to the students from UoS, students from VCU rated the e-compendiums more positively in most aspects of learning. Exceptions were for found for statements regarding the value of being introduced to the topic and for applying new knowledge. On these statements, VCU students scored significantly lower than UoS students. The UoN students found the e-compendiums to be more important for learning engagement compared to UoS students. No difference was found in any other aspects of learning. Odds ratios from logistic regression analysis for the differences between UoN and UoS as well as between VCU and UoS are shown in Table 4.

**Table 4:** Odds ratios (and 95 % confidence interval) from logistic regression analysis identifying associations between agree and strongly agree for learning and institutions by gender and age. Reference institution is University of Stavanger

Strongly agree or agree that the e-compendiums were useful for	University of Nottingham		Valencia Catholic University	
	OR (95 % CI)	*p*-value	OR (95 % CI)	*p*-value
Being introduced to the topic	0.73 (0.22–2.4)	0.603	0.18 (0.094–0.35)	< 0.001
Reinforcing my knowledge	3.4 (0.69–16.2)	0.131	3.4 (1.46–8.2)	0.005
Retaining my knowledge	2.0 (0.56–7.3)	0.286	3.9 (1.77–8.1)	< 0.001
Applying new knowledge	0.88 (0.36–2.2)	0.790	0.4 (0.21–0.57)	< 0.001
Focusing on essential parts	1.69 (0.73–3.9)	0.222	2.5 (1.48–4.21)	<0.001
Meeting the requirement of the course	1.2 (0.52–2.7)	0.626	2.6 (1.49–4.5)	<0.001
Self-assessment	1.9 (0.50–7.1)	0.350	3.5 (1.64-7. 2)	<0.001
Increasing my confidence	1.23 (0.43–3.6)	0.697	2.1(1.05-4.0)	0.034
Increasing my motivation	1.41 (0.63–3.1)	0.416	2.7 (1.64–4.5)	< 0.001
Enjoyment of learning	1.84 (0.90–3.7)	0.090	8.3 (4.9–13.9)	< 0.001
Being more engaged	3.1 (1.44–6.8)	0.004	9.2(5.33–15.7)	< 0.001

## Discussion

This is one of a few studies that has explored usability and value for learning of a shared repurposed e-learning resource in nurse education across three European universities. Despite being originally developed for students in Norway, the e-compendiums were in most aspects of learning rated more positively by students in Spain. Further, the UK students did not value the e-compendiums any less than students at the Norwegian study site; in fact, they found them to be more important for learning engagement. One explanation for this positive response from all three intuitions might be linked to the interdisciplinary approach in the development of the adapted e-compendiums. Academic teaching staff and technical staff from all three institutions were included in workshops, transnational meetings and feedback loops. The use of a transparent collaborative digital working platform laid the foundation for open and visible communication in the project group [16]. As lack of support from technical experts is often described as a barrier for e-learning development [21, 22], our close interdisciplinary collaboration might have played an important role in the quality of the solution.

Another explanation for the positive results might be the emphasis on user involvement in the current project. Due to policy initiatives, students should become involved as co-creators of their own learning, and student involvement has increasingly been seen as important for health education improvement [23, 24]. In the current project, student representatives from all three universities participated in all phases of the adaptation and translation process. The goal for this involvement was to place students’ needs at the centre of the design process, based on the view of students as a knowledgeable and critical partner in learning [25]. Their critical feedback guided the further development of the e-compendiums. For instance, based on students’ feedback, more images and interactive elements as well as the use of audio were included in the e-compendiums.

Interestingly, students at the VCU were more enthusiastic about the e-compendiums than students from the UoS. In fact, students at VCU valued the learning tool up to 9 times more highly for some aspects of learning compared to students at the UoS. With regard to the validity of our results, it is important to emphasize that the three universities did not differ regarding attitudes towards e-learning in general in terms of confidence in the use of digital devices. The reasons for the higher scores among students at the VCU are not clear. It has been claimed that study participants from different cultures might respond differently on Likert scales, such as in their willingness to select extreme responses [26]. However, this has mostly been an issue when reporting strong emotional matters and therefore might not be the case in our study, which focused on learning-oriented issues [27]. Another explanation for the difference might be that the UoS and the UoN have longer traditions of using e-learning approaches in nurse education compared to the VCU, which has adopted a more traditional learning approach. Thus, one could assume that the e-compendiums represented an exciting, novel approach for the students at the Spanish study site.

For the total group, the e-compendiums were especially valued for reinforcing and retaining knowledge. Furthermore, our findings indicate that visual and interactive elements in the e-compendiums were more appreciated by younger students than older ones. This might be explained by the fact that younger students have grown up with new technology and are described as “digital scholars” [28]. They use technology of all forms for research, communication, and data processing and, consequently, accept modernized pedagogical approaches when entering higher education. Our study also indicated some differences between male and female students in terms of usability of the e-compendiums. For example, female students more often reported that the audio element—being able to listen to the content as a podcast—was important than male students. The advantages of audio learning material have been previously reported due to the efficiency of listening while doing other time-consuming activities, such driving, walking, or doing housework [29]. Whether or not the aspects of multitasking are linked to gender is not clear. However, our results support the conclusion that e-learners seem to differ in their preferences in relation to aspects beyond age [29].

The present study has some limitations. Despite including a large number of participants, the participants were not equally distributed across the three institutions. Due to practical issues, UoN included a smaller number of participants than VCU and UoS. Therefore, the interpretation of the results comparing UoN and UoS should be done with caution. Furthermore, due to the lack of an existing suitable questionnaire being specific and sensitive enough to capture the value and usability of the specific e-learning tool in our study, we adapted a questionnaire used for evaluating RLOs. Although this adapted version of the questionnaire was not validated or pre-tested, it was adapted within the context of our study. This included perspectives from researchers, teachers, technicians, and students. The adapted version was perceived to be relevant for capturing the relevant aspects of the value and usability of the e-learning tool. Hence, the acceptability, feasibility, and relevance of the questionnaire were ensured.

Based on the experiences from the current project, sharing of learning material across institutions from different countries required more work than assumed. It was never assumed to be a simple language translation exercise but there was considerable adaptation needed in terms of culture, practical guidelines and pedagogical traditions. For instance, the nutrition topic required rigorous adaption as this strongly linked to culture [17]. To our knowledge, there are no guidelines for translation of learning material across cultures. However, for future work we would suggest a need for building rigorous guidelines similar to those required for translation of cross-culture questionnaire instruments. Systematic use of activities such as “back translation” and “face validity” are key steps in instrument translation and similar standardised protocols could be of help also in the translation of pedagogical material [30].

## Conclusions

In conclusion, the current study has provided valuable insights into the sharing of e-learning material. First of all, sharing of learning material across countries in the context of nursing education seems feasible and useful. The current study showed that the UoN and VCU students accepted the e-compendiums in line with the students where these materials were originally developed. Global pedagogical projects in higher education is a European priority and findings from the current study might encourage future international pedagogical projects. However, as considerable adaptation is required in terms of culture, practical guidelines and pedagogical culture, we argue that adaptation and translation processes that include multicultural and multidisciplinary project members should be recommended for future projects.

## Supplementary Information

**Additional file 1.**

**Additional file 2.**

## Data Availability

The dataset used and analysed during the current study is available from the corresponding author on reasonable request.

## Abbreviations

UK: United Kingdom.
UoS: University of Stavanger.
UoN: University of Nottingham.
VCU: Valencia Catholic University.
RLO-CETL: Centre for Excellence in Teaching and Learning Reusable Learning Object evaluation questionnaire.
RLO: Re-usable learning objects.
SPSS: Statistical Package for Social Sciences.
