# Supplementary figures and images for: Usability and value of a digital learning resource in nursing education across European countries: a cross-sectional exploration
Source: BMC Nurs. 2021 Sep 6;20:161. doi: 10.1186/s12912-021-00681-5 (PMC8419383; doi:10.1186/s12912-021-00681-5)

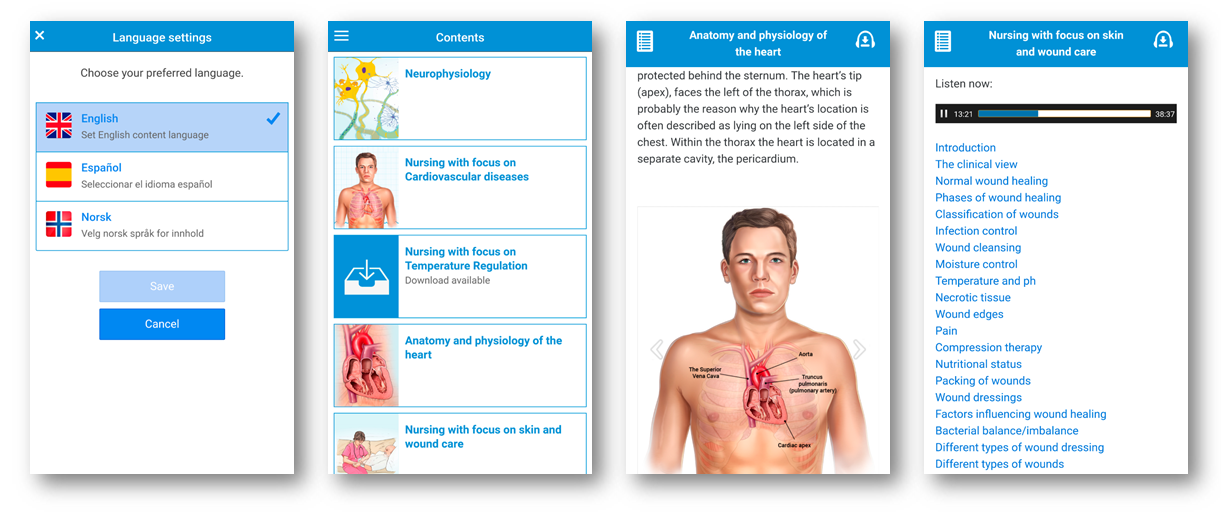

Supplement: Supplementary file 1 — Additional file 1. [file 12912_2021_681_MOESM1_ESM.png]

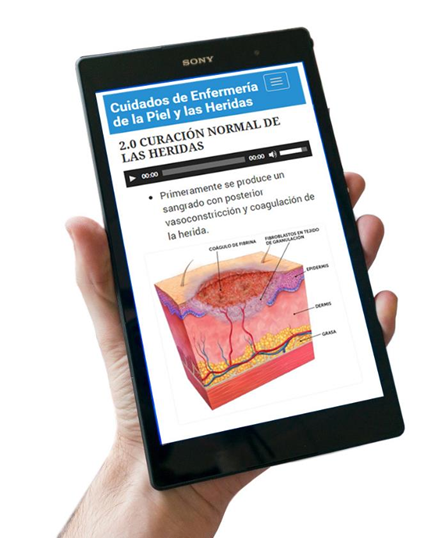

Supplement: Supplementary file 2 — Additional file 2. [file 12912_2021_681_MOESM2_ESM.png]
